# Integration of Transcriptomics With Interpretable Artificial Intelligence for Identifying Molecular Signatures of Physiological Stress in Sleep Deprivation

**DOI:** 10.1111/jcmm.71211

**Published:** 2026-05-29

**Authors:** Kun Wang, Qiang Zong, Chengcheng Wang, Peng Wang, Zhenhao Shuai, Min Wu, Yuming Peng, Junying Zhou, Jianwei Shuai, Fangfu Ye, Aimin Wu, Yanyan Zheng

**Affiliations:** ^1^ The Wenzhou Third Clinical Institute Affiliated Wenzhou Medical University Oujiang Laboratory (Zhejiang Lab for Regenerative Medicine, Vision and Brain Health) Wenzhou Institute UCAS Wenzhou Zhejiang China; ^2^ Department of Neurology, West China Hospital Sichuan University Chengdu Sichuan China; ^3^ Department of Public Health and Health Management, Clinical College of Anhui Medical University Hefei Anhui China; ^4^ Department of Computer Science and Technology Xiamen University Xiamen Fujian China; ^5^ Department of Geriatrics Central Hospital of Karamay Xinjiang China; ^6^ Sleep Medicine Centre, West China Hospital Sichuan University Chengdu Sichuan China; ^7^ Department of Orthopaedics, Key Laboratory of Orthopaedics of Zhejiang Province The Second Affiliated Hospital and Yuying Children's Hospital of Wenzhou Medical University Wenzhou China

**Keywords:** feature genes, immune infiltration, machine learning, *S100A3*, sleep deprivation

## Abstract

Sleep deprivation induces systemic physiological stress accompanied by transcriptomic remodelling and immune dysregulation, yet objective molecular indicators for its assessment remain insufficient. This study integrated blood transcriptomic analysis with an interpretable machine learning framework to identify and validate candidate molecular signatures associated with sleep deprivation and their potential relevance to insomnia. Publicly available Gene Expression Omnibus datasets were used to construct an acute sleep deprivation training cohort, an independent sleep deprivation validation cohort, and a chronic insomnia validation cohort. Differentially expressed genes were first identified, followed by feature selection using six machine learning algorithms and Shapley additive explanations to improve model interpretability. Immune cell composition was inferred using CIBERSORT, and associations between candidate genes and immune cell subsets were further evaluated. Twenty‐five differentially expressed genes were identified in the training cohort, from which eight high‐priority candidate genes were selected by the interpretable machine learning framework. Among them, S100A3 showed consistent discriminatory performance across the training cohort, the independent sleep deprivation cohort, and the insomnia cohort, whereas VEGFB exhibited notable diagnostic potential, particularly in insomnia. Immune infiltration analysis indicated that sleep deprivation was associated with altered peripheral immune composition, including reduced resting natural killer cells and activated dendritic cells, together with changes in regulatory and naïve immune cell populations. Expression levels of S100A3 and VEGFB were significantly correlated with specific immune cell subsets, suggesting a link between these molecular signatures and stress‐related immunomodulation. These findings identify S100A3 as a robust candidate biomarker shared by acute sleep deprivation and chronic insomnia, while VEGFB may reflect chronic metabolic or inflammatory adaptation. The proposed interpretable transcriptomic‐machine learning framework provides a non‐invasive strategy for discovering molecular indicators of sleep‐related physiological stress and may support future risk stratification in sleep medicine.

**Abbreviations**
|log₂FC|: Absolute log₂‐fold change
|NES|: Absolute normalised enrichment score
AUC: Area under the curve
AUROC: Area under the receiver operating characteristic
DAMPs: Damage‐associated molecular patterns
DAVID: Database for annotation, visualisation, and integrated discovery
DEGs: Differentially expressed genes
GEO: Gene expression omnibus
GO‐BP: Gene ontology biological process
GO: Gene ontology
GSEA: Gene set enrichment analysis
MLP: Multilayer perceptron
NB: Naive bayes
NK: Natural killer
PCA: Principal component analysis
RF: Random forests
ROC: Receiver operating characteristic
SD: Sleep deprivation
SHAP: SHapley additive exPlanations
SVM: Support vector machine
Tregs: Regulatory T cells
XGBoost: Extreme gradient boosting

## 1 Introduction

Sleep, a physiological process fundamental to homeostasis, cognitive function, and overall health, plays a pivotal role in regulating energy metabolism, memory consolidation, and cellular repair through the neuroendocrine–immune network [1, 2]. However, amid globalisation and modernisation, accelerated lifestyles and widespread increases in psychosocial stress have elevated sleep disorders into a severe global public health challenge. An epidemiological study conducted in the Netherlands revealed that approximately 43.2% of adults reported insufficient sleep duration [3]. Chronic sleep deprivation is strongly associated with the development of major chronic diseases. Epidemiological studies have confirmed its link to cardiovascular risk, with a meta‐analysis showing that reduced sleep duration increases cardiovascular disease risk [4]. At the metabolic level, insufficient sleep is associated with all components of metabolic syndrome, including obesity, type 2 diabetes, and hypertension [5, 6, 7]. Another meta‐analysis reported a 1.61‐fold greater risk of hypertension in individuals sleeping < 5 h than in those sleeping 7 h [8]. With respect to neurocognitive function, sleep is essential for central nervous system health because it facilitates the clearance of metabolic byproducts; disruption of this process elevates the risk of depression and impairs learning and memory consolidation [9, 10].

From the macro level of clinical phenotypes to the micro molecular level, modern molecular biology research has revealed that sleep deprivation involves not only a passive absence of rest but also an actively triggered, intense physiological stress response. This response induces extensive and profound molecular landscape remodelling throughout the body. Its core pathophysiological alterations unfold primarily along two intertwined pathways. The first involves profound disruptions at the transcriptomic and epigenetic levels. Studies have confirmed that abnormal expression patterns of core circadian rhythm genes (such as CLOCK and PER2) can interfere with metabolic and neural functions [11]. Moreover, multiple epigenetic regulatory mechanisms—including DNA methylation, histone modifications, and noncoding RNAs—have been shown to play crucial roles in sleep deprivation‐induced cognitive impairment, mediating persistent alterations in gene expression patterns [12].

The second critical pathway is characterised by immune system dysregulation and the establishment of chronic low‐grade inflammation. Sleep serves as a critical time window for calibrating innate and adaptive immune functions. Nevertheless, sleep deprivation severely disrupts this finely tuned equilibrium, plunging the body into a chronic, systemic state of low‐grade inflammation characterised by markedly elevated levels of proinflammatory cytokines in the circulation [13]. Consequently, this chronic inflammatory state constitutes a key pathophysiological foundation driving the progression of multiple diseases, including atherosclerosis, insulin resistance, and neurodegenerative disorders.

Despite considerable progress in elucidating the molecular underpinnings of sleep deprivation, its clinical assessment remains highly challenging. Current evaluations of sleep deprivation largely depend on subjective self‐report instruments, which are inherently influenced by recall bias and psychological states, thereby showing limited concordance with objective physiological measures. In addition, objective approaches such as polysomnography, although regarded as the gold standard for sleep assessment, are constrained by high cost, technical complexity, and poor feasibility in large‐scale population studies, and they provide little information on the underlying pathophysiological mechanisms. Consequently, there is an urgent need to establish molecular biomarkers capable of objectively and quantitatively reflecting the severity of sleep deprivation, thereby enabling accurate risk stratification and advancing clinical translation in sleep medicine [14].

Computational modelling and network medicine approaches are becoming indispensable. Advanced analytical strategies have proven highly effective in deciphering high‐dimensional biological data—from proteomics to single‐cell transcriptomics—to reveal hidden associations in complex biological processes [15, 16, 17, 18]. Furthermore, the convergence of large‐scale omics with advanced analytical frameworks, such as multimodal fusion, heterogeneous graph networks, and Mamba architectures, is transforming clinical practice, enabling biomarker discovery across a wide spectrum of diseases [19, 20, 21]. However, to ensure clinical trust, establishing interpretable AI frameworks is crucial. Machine learning algorithms, such as support vector machines and random forests, have been widely employed in biomarker research [22, 23], and feature selection approaches have shown increasing utility in sleep research [24].

Therefore, this study proposes an innovative feature selection framework that integrates bioinformatics analysis with interpretable machine learning algorithms. Our primary aim is to systematically identify candidate genes strongly associated with sleep deprivation and to rigorously validate their role as signature genes in an independent sleep deprivation cohort. We further aim to investigate the broader significance of these markers in the context of chronic insomnia. Although clinically distinct, acute sleep deprivation and insomnia converge on fundamental molecular pathways, including the activation of neuroinflammatory signalling and the dysregulation of the immune system [25]. This shared biology provides a strong rationale for examining whether biomarkers of acute sleep deprivation are similarly dysregulated in chronic insomnia. Therefore, we extended our validation to an independent insomnia cohort, aiming to assess the potential of these genes as shared molecular markers.

## 2 Materials and Methods

**FIGURE 1:**
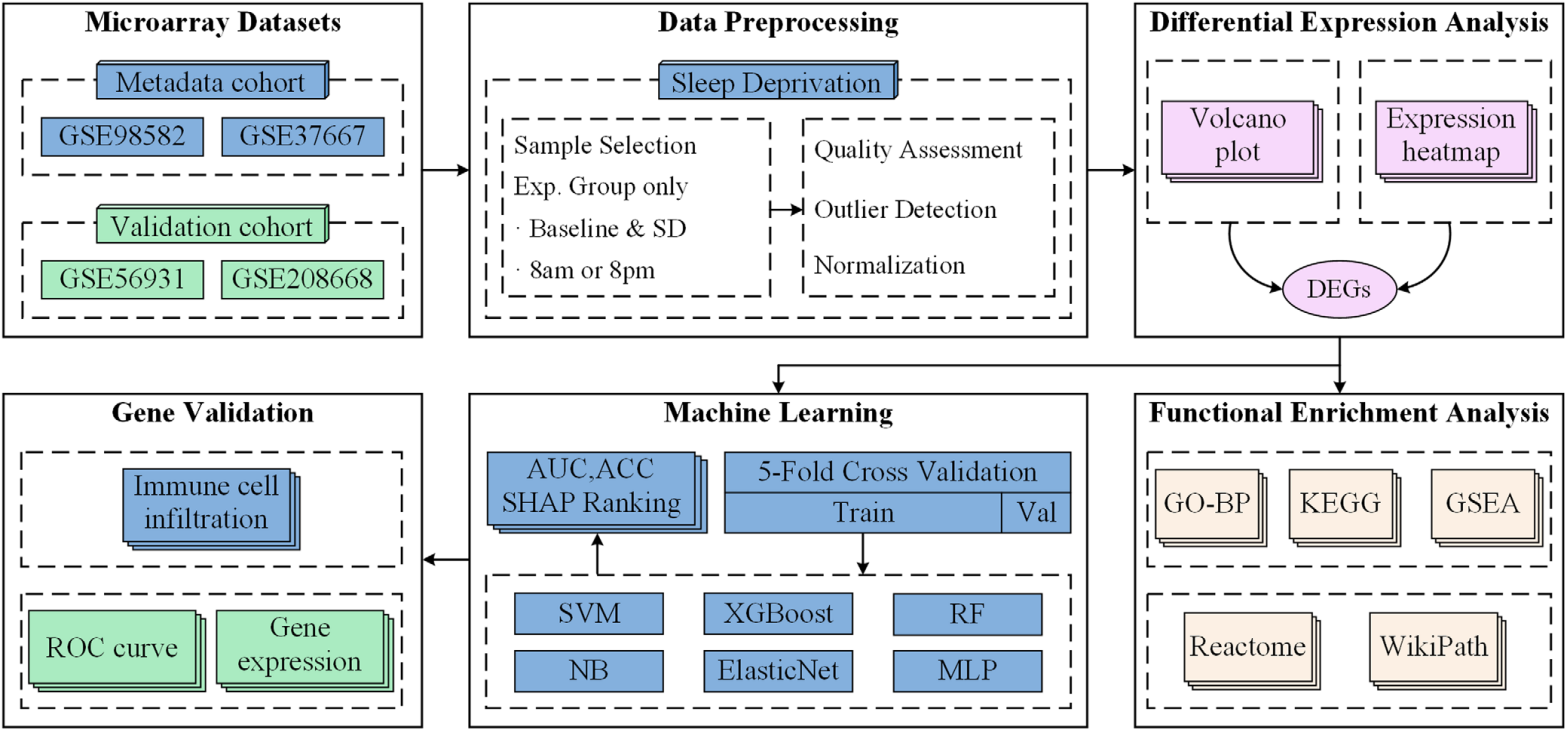
Research flowchart. This diagram illustrates the comprehensive workflow encompassing data acquisition, preprocessing, differential gene expression analysis, functional enrichment analysis, machine learning feature selection, diagnostic model construction, and validation analysis.

The overall workflow of this study is illustrated in Figure 1, encompassing key steps such as data acquisition and preprocessing, differential expression analysis, functional enrichment analysis, machine learning and feature selection, candidate gene validation, and immune infiltration analysis.

### 2.1 Data Acquisition and Preprocessing

Four publicly available transcriptomic microarray datasets were retrieved from the GEO database to define the analytical cohorts. All data processing and subsequent analyses were conducted in the R environment. The training cohort was generated by combining two acute sleep deprivation datasets: GSE98582 [26] and GSE37667 [27]. For independent validation, an additional sleep deprivation dataset (GSE56931 [28]) and a chronic insomnia dataset (GSE208668 [29]) were employed. The baseline characteristics of all the cohorts are detailed in Table S1. The insomnia cohort, which included elderly patients and age‐matched controls, was specifically incorporated to assess the broader clinical relevance of biomarkers in the context of chronic sleep disorders.

A standardised preprocessing pipeline was applied to ensure data quality and comparability. First, probe IDs in each dataset were mapped to official gene symbols via the corresponding platform annotation files. Hierarchical clustering was then performed to identify and exclude outlier samples. The gene expression values were subsequently normalised via quantile normalisation using the *normalizeBetweenArrays* function of the *limma* package. Finally, to reduce interdataset variability, batch effects across datasets were corrected via the *ComBat* function of the *sva* package.

Additionally, a normal human circadian rhythm dataset (GSE48113 [30]) was acquired from the GEO database to serve as a negative control. The expression stability of candidate biomarkers and immune infiltration profiles across physiological states was evaluated using one‐way analysis of variance (ANOVA) and CIBERSORT, respectively.

### 2.2 Differential Expression Analysis

DEGs were identified from the integrated training cohort via the *limma* package, which applies linear modelling and empirical Bayesian methods to stabilise variance estimates and thereby improve statistical power in studies with limited sample sizes.

Genes were defined as differentially expressed when both an adjusted *p* value < 0.05 (adjusted by the Benjamini‐Hochberg false discovery rate method) and absolute log_2_‐fold change (|log_2_FC|) ≥ 0.3 were met. We specifically adopted the |log_2_FC| threshold because sleep deprivation, as a physiological stressor, typically induces widespread yet modest transcriptional perturbations. This more sensitive cutoff was therefore considered critical for capturing subtle but biologically meaningful changes that might otherwise be missed under more stringent criteria.

### 2.3 Functional Enrichment Analysis

The potential biological functions of the identified DEGs were explored through pathway enrichment analysis via Database for Annotation, Visualisation, and Integrated Discovery (DAVID). This analysis integrated multiple resources, including gene ontology biological process (GO‐BP), KEGG, Reactome, and WikiPathways, with statistical significance defined as *p* < 0.05.

In addition, pathway‐level insights were further examined by performing gene set enrichment analysis (GSEA) with the *clusterProfiler* package, thereby complementing DEG‐based approaches and reducing potential biases associated with arbitrary differential expression thresholds. In this analysis, all detected genes were ranked according to their log_2_FC values, and enrichment was assessed against predefined KEGG gene sets. Significant enrichment was determined on the basis of *p* < 0.05, FDR < 0.25, and absolute normalised enrichment score (|NES|) > 1.

### 2.4 Machine Learning and Feature Selection

A multimodel machine learning framework was constructed to systematically identify the most predictive biomarkers from the pool of DEGs. Six representative algorithms were incorporated to capture diverse modelling strategies and minimise biases inherent to any single approach: Support vector machine (SVM), extreme gradient boosting (XGBoost), random forests (RF), naive bayes (NB), Elastic Net, and multilayer perceptron (MLP). All the models were trained and evaluated under a fivefold cross‐validation scheme with a global random seed set to ensure reproducibility. Model performance was comprehensively evaluated via multiple metrics, including accuracy, the area under the receiver operating characteristic (AUROC) curve, specificity, precision, recall, and the F1 score.

Model interpretability was enhanced via the SHAP method implemented in the *fastshap* package. To concretely demonstrate the interpretability of our black‐box models, SHAP was utilised to explicitly quantify the exact contribution of each gene to the classification output. On the basis of game theory, SHAP assigns each feature a marginal contribution value that reflects its influence on model output. In this study, this interpretability is visually and quantitatively demonstrated through feature importance rankings. Rather than merely providing a binary prediction, this approach transparently reveals which specific candidate biomarkers drive the model's decision to classify a sample as sleep‐deprived vs. normal, and by what magnitude. The global SHAP values for each gene were computed across all six models. To enable direct comparison across algorithms with varying output scales, the absolute SHAP values for each gene were normalised to a [0, 1] range using Min‐Max scaling:

$\text{Normalized}\_\text{Importance}=\frac{|{\text{SHAP}}_{\text{gene}}|-\min\left(\text{SHAP}\right)}{\max\left(\text{SHAP}\right)-\min\left(\text{SHAP}\right)}$

The global SHAP values for each gene were computed across all six models, normalised, and aggregated to generate a composite importance score. Genes with a total normalised SHAP value ≥ 2 were considered robust features and were retained as core candidate genes for further validation.

### 2.5 Candidate Gene Validation

The diagnostic efficacy of the candidate genes was assessed in both the training and validation cohorts through single‐gene receiver operating characteristic (ROC) curve analysis. This analysis was conducted with the *pROC* package in R to evaluate the discriminative ability of each gene between the experimental and control groups. The area under the curve (AUC) served as the primary performance metric, with values greater than 0.7 interpreted as evidence of good predictive performance.

### 2.6 Immune Infiltration Analysis

The impact of sleep deprivation on the peripheral immune microenvironment was evaluated by inferring the relative abundance of 22 immune cell subpopulations from bulk gene expression data. This deconvolution was performed via the CIBERSORT algorithm, implemented through the *immunedeconv* package, with the LM22 signature matrix. The statistical significance of each sample's deconvolution was determined via 1000 permutations.

Differences in immune cell proportions between the sleep‐deprived and control groups, as well as between the insomnia and control groups, were assessed via the Wilcoxon signed‐rank test. Spearman correlation analysis was subsequently conducted to investigate potential regulatory relationships between the expression levels of candidate genes and the abundance of various immune cell types.

### 2.7 Statistical Analysis

All the statistical analyses were conducted in the RStudio environment via R version 4.3.0. Comparisons of continuous variables between two groups were performed via either Student's *t*‐test or the Wilcoxon signed‐rank test, depending on the normality of the data distribution. Differences in immune cell infiltration proportions were specifically assessed via the Wilcoxon test because of the nonparametric nature of the CIBERSORT output. Associations between gene expression and immune cell abundance were quantified via Spearman's correlation coefficient. Unless otherwise indicated, all the statistical tests were two‐tailed, and a *p* < 0.05 was considered statistically significant.

## 3 Results

### 3.1 Data Preprocessing and Quality Control

**FIGURE 2:**
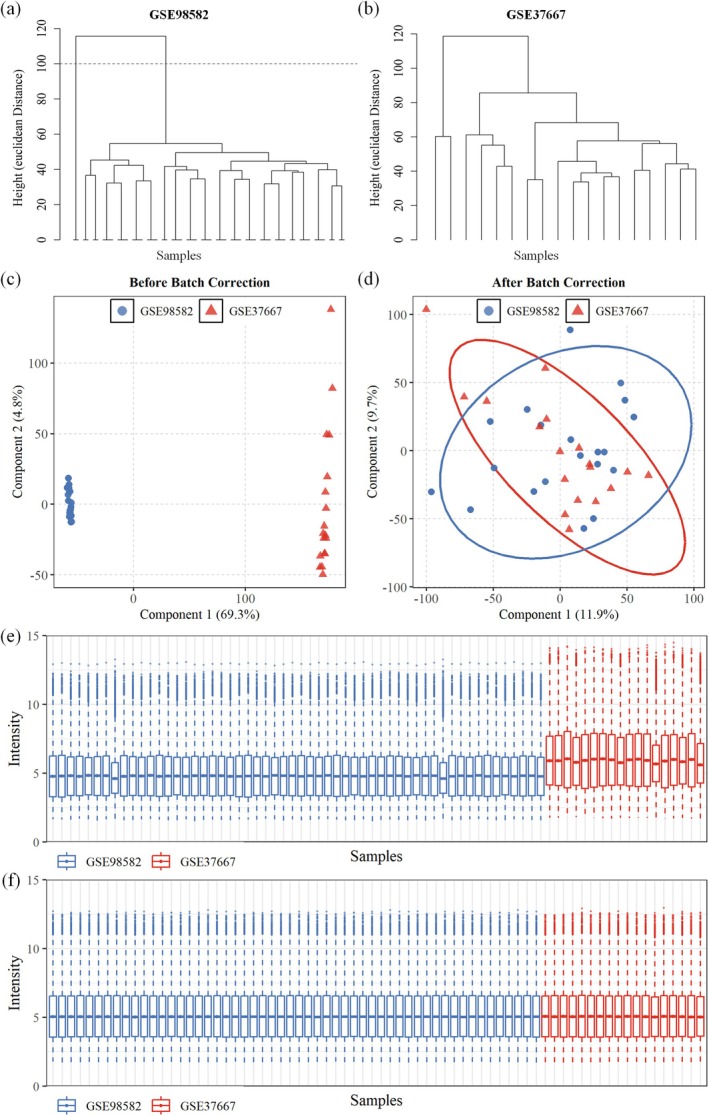
Data preprocessing and quality control. (a, b) Hierarchical clustering of the GSE98582 and GSE37667 datasets; (c, d) PCA plots before and after batch effect correction; (e, f) Boxplots showing gene expression distributions before and after quantile normalisation and batch effect removal.

Following sample screening and quality control, the final training cohort comprised 72 blood samples, consisting of equal numbers of samples from the sleep deprivation (*n* = 36) and matched control (*n* = 36) groups. The two independent validation sets included a sleep deprivation cohort with 27 samples and a chronic insomnia cohort with 42 samples. As part of the initial data quality assessment, hierarchical clustering was performed on the two training datasets, GSE98582 and GSE37667. The resulting dendrogram for GSE98582 identified significant outlier samples (Figure 2a), whereas no significant outlier samples were identified for GSE37667 (Figure 2b).

Prior to data integration, principal component analysis (PCA) revealed that samples clustered primarily by their dataset of origin rather than by biological condition, which indicated the presence of significant batch effects (Figure 2c). After application of the *ComBat* algorithm, these batch effects were effectively mitigated, leading to a more coherent sample distribution in the PCA plots (Figure 2d). Concurrently, quantile normalisation harmonised the expression distributions across all samples, as shown in the postprocessing boxplots (Figure 2e,f). These observations indicated that the integrated dataset was appropriate for subsequent differential expression analysis.

### 3.2 Identification of Differentially Expressed Genes

**FIGURE 3:**
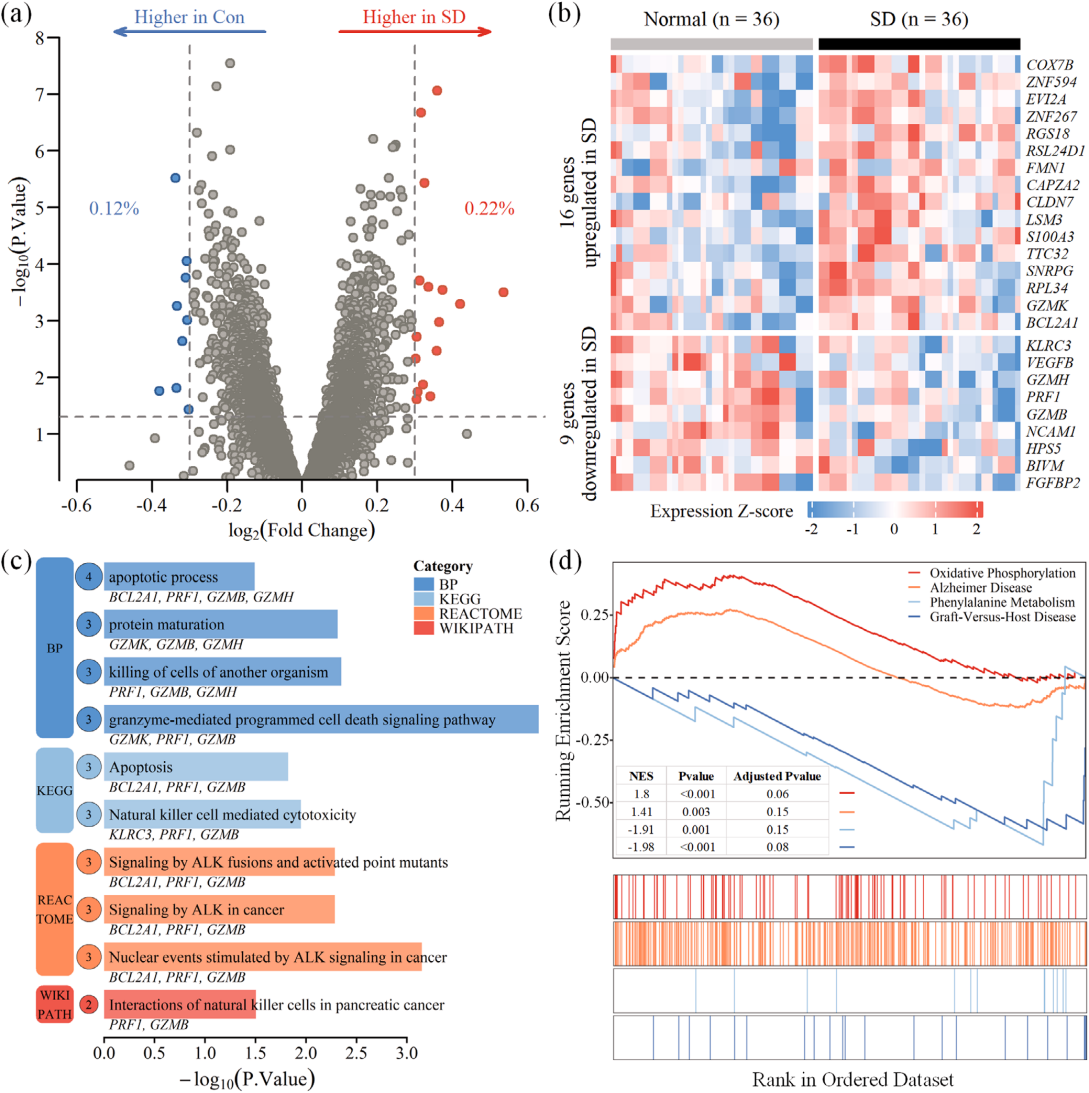
Differentially expressed genes and pathway analysis. (a) Volcano plot of DEGs between the sleep deprivation and control groups (*p* < 0.05, ∣log2 FC∣ ≥ 0.3); (b) Heatmap of 25 DEGs showing distinct expression patterns; (c) Barplots of GO‐BP, KEGG, Reactome, and WikiPathways enrichment; (d) GSEA curves indicating pathways enriched in the sleep deprivation (red) and control (blue) groups.

Based on the predefined thresholds, a total of 25 significant DEGs were identified in the training cohort (see Table S2 for details). Among these genes, 16 genes were upregulated, and 9 genes were downregulated in the SD group compared with the control group. The distribution of these DEGs with respect to statistical significance and fold change is illustrated in a volcano plot (Figure 3a).

Hierarchical clustering was subsequently performed to assess the collective discriminatory capacity of the 25 DEGs. The resulting heatmap demonstrated that the expression patterns of the DEGs effectively segregated the samples into two distinct clusters corresponding to the sleep deprivation and control groups (Figure 3b).

### 3.3 Functional Enrichment Analysis

Pathway and GO enrichment analyses were performed to investigate the biological functions of the 25 identified DEGs. At the GO biological process level, the DEGs were significantly enriched in immune‐ and cell death‐related pathways, including the granzyme‐mediated programmed cell death signalling pathway and apoptotic process (Figure 3c). Similarly, pathway analysis revealed significant involvement in NK cell‐mediated cytotoxicity and apoptosis.

To provide a broader perspective on transcriptomic alterations, GSEA was conducted on the entire ranked gene list. The GSEA results revealed significant upregulation of pathways associated with metabolic stress and neurodegeneration, including oxidative phosphorylation and Alzheimer's disease, in the sleep deprivation group. Conversely, pathways such as phenylalanine metabolism and graft‐vs.‐host disease were significantly enriched in the control group (Figure 3d).

### 3.4 Machine Learning and Feature Selection

**FIGURE 4:**
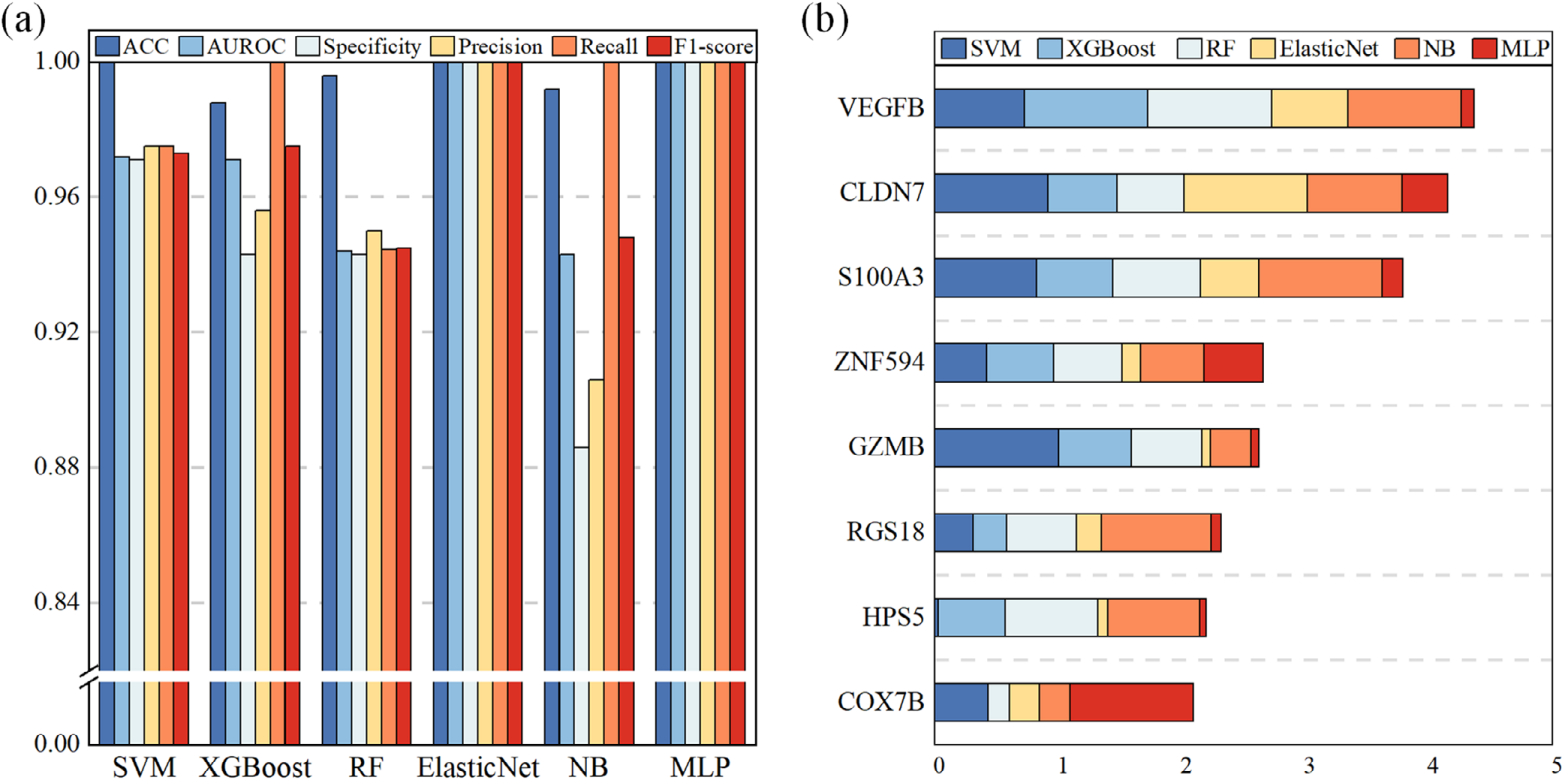
Machine learning and feature selection. (a) Performance of six machine learning models in fivefold cross‐validation, evaluated by accuracy, AUC, specificity, precision, recall, and F1 score; (b) SHAP bar plots showing feature importance scores of eight candidate genes across models.

Six machine learning models were constructed using the 25 DEGs as input features and subsequently evaluated through a fivefold cross‐validation scheme. All the models exhibited high classification performance in distinguishing sleep‐deprived samples from control samples (Figure 4a). The optimal parameters and detailed performance Metrics for each model are provided in Tables S3 and S4, respectively. The Elastic Net and MLP models achieved the highest performance, with perfect accuracy and AUROC (1.00), whereas the remaining models also performed robustly: RF (accuracy = 0.96, AUROC = 0.99), SVM (accuracy = 0.95, AUROC = 0.98), XGBoost (accuracy = 0.95, AUROC = 0.99), and NB (accuracy = 0.94, AUROC = 0.98).

On the basis of SHAP analysis and the predefined selection criterion, a final set of eight candidate genes with the highest predictive importance was identified (Figure 4b). The complete SHAP values for all genes across the six models are listed in Table S5. These genes were ranked in descending order according to their total SHAP values as follows: *VEGFB*, *CLDN7*, *S100A3*, *ZNF594*, *GZMB*, *RGS18*, *HPS5*, and *COX7B*.

### 3.5 Validation of Candidate Genes

**FIGURE 5:**
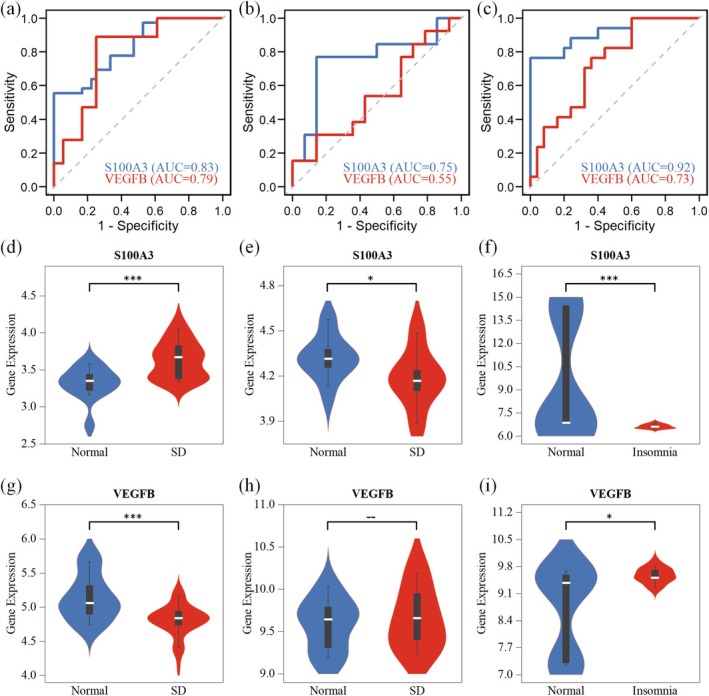
Validation of candidate genes. (a–c) ROC curves for evaluating the diagnostic performance of candidate genes in the training, sleep deprivation validation, and insomnia cohorts. (d–i) Boxplots comparing the expression of *S100A3* and *VEGFB* between the case and control groups across datasets (nonparametric test).

The diagnostic performance of the eight candidate genes was assessed via ROC analysis across the three cohorts, which identified *S100A3* and *VEGFB* as the most robust performers. *S100A3* exhibited consistent and strong discriminatory power across all datasets, achieving an AUC of 0.83 in the training cohort, maintaining robust performance in the sleep deprivation validation cohort, with an AUC of 0.75, and demonstrating excellent diagnostic potential in the insomnia cohort, with an AUC of 0.92. *VEGFB* also showed notable classification ability in the training and insomnia cohorts; however, its discriminatory power was not significant in the sleep deprivation validation cohort (Figure 5a–c). The remaining six candidate genes did not consistently reach effective diagnostic performance across the validation cohorts (Figure S1).

Consistent with the ROC analysis, Wilcoxon rank‐sum tests confirmed that *S100A3* expression was significantly altered in the case groups of all three cohorts. *VEGFB* expression was significantly different in the training and insomnia cohorts but not in the sleep deprivation validation cohort (Figure 5d–i).

### 3.6 Analysis of Immune Cell Infiltration

**FIGURE 6:**
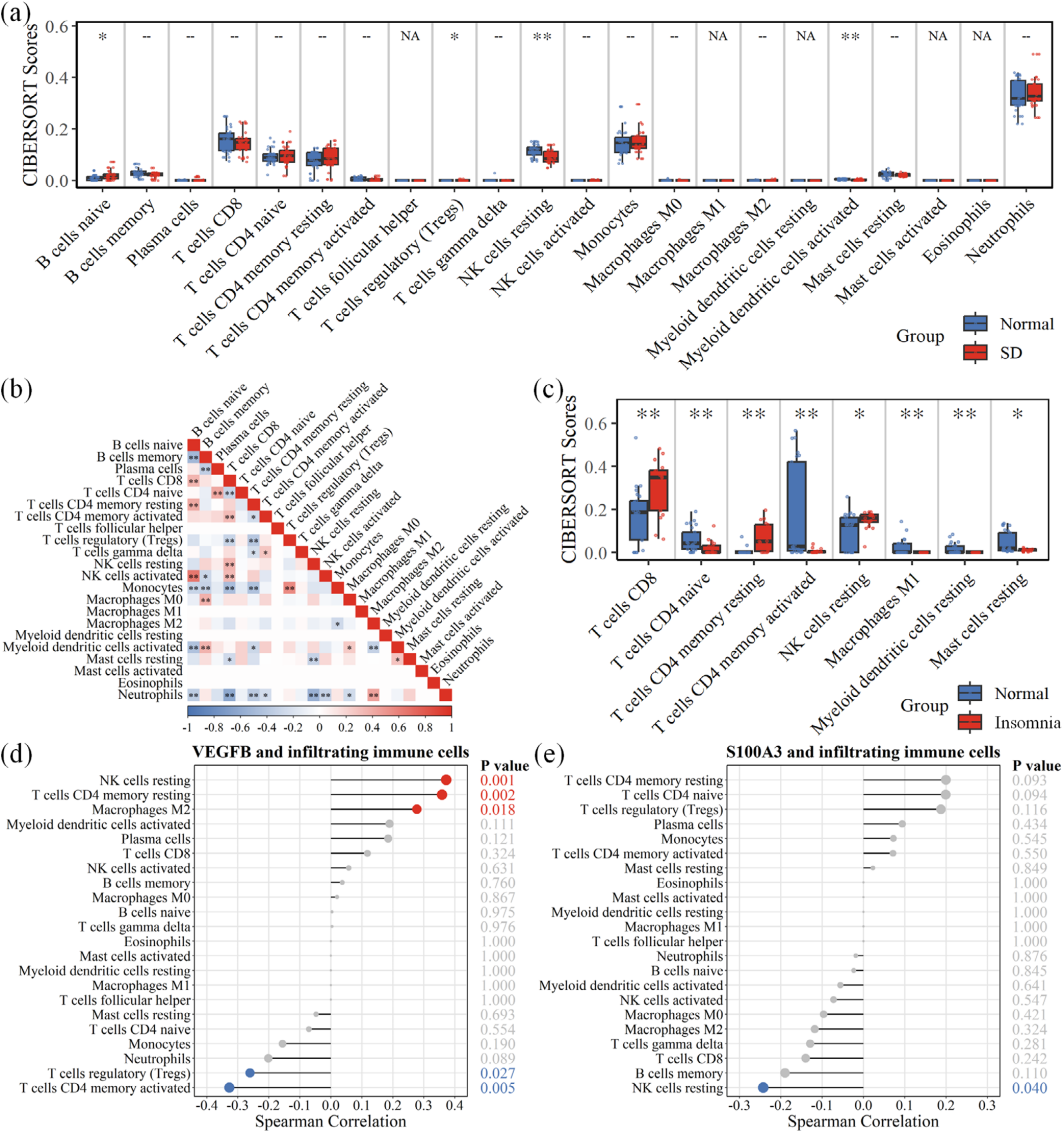
Immune infiltration and gene associations. (a) Boxplots showing differences in 22 immune cell subsets between the sleep deprivation and control groups via CIBERSORT (Wilcoxon test); (b) Pearson correlation heatmap of immune cells; (c) Boxplots showing differences in 22 immune cell subsets between the insomnia and control groups; (d, e) Spearman correlation plots between *S100A3* or *VEGFB* expression and immune cell proportions, with the dot size indicating correlation strength and colour intensity representing significance.

The results of the Wilcoxon rank‐sum test indicated that the sleep‐deprived state significantly altered the immune cell composition in peripheral blood (Figure 6a). Compared with the healthy control group, samples from the sleep deprivation group showed a significant increase in naive B cells and a notable increase in Treg cells. Conversely, the proportions of two crucial effector immune cells were significantly reduced: Resting NK cells and activated myeloid dendritic cells.

An immune cell correlation heatmap revealed complex interrelationships, such as a positive correlation between naive B cells and CD8+ T cells and a negative correlation between regulatory T cells (Tregs) and CD8+ T cells (Figure 6b). In the insomnia dataset, immune infiltration analysis revealed differential proportions of CD8+ T cells, naive CD4+ T cells, resting memory CD4+ T cells, activated memory CD4+ T cells, resting NK cells, M1 macrophages, resting myeloid dendritic cells, and resting mast cells compared with those in the control group (Figure 6c). Further correlation analysis revealed that the expression level of *VEGFB* was positively correlated with that of resting NK cells, resting memory CD4 T cells, and M2 macrophages but was negatively correlated with that of Tregs and activated memory CD4 T cells. The expression of *S100A3* was negatively correlated with that of only resting NK cells (Figure 6d,e).

### 3.7 Validation of Biomarker Specificity in a Normal Circadian Rhythm Cohort

Excluding potential confounding effects of natural circadian rhythms, we evaluated the stability of the identified biomarkers and immune infiltration patterns in an independent negative control cohort. One‐way ANOVA revealed that *S100A3* expression remained remarkably stable across a 24‐h normal sleep–wake cycle, with no statistically significant fluctuations observed between different time points (*p* = 0.413; Figure S2a). Furthermore, the CIBERSORT analysis of GSE48113 revealed a distinct circadian pattern of immune cell trafficking. In this normal rhythm cohort, we observed significant physiological fluctuations in certain subsets, including memory B cells (*p* < 0.01), activated myeloid dendritic cells (*p* < 0.01), follicular helper T cells (*p* < 0.05), and monocytes (*p* < 0.05), alongside an increase in neutrophils (*p* < 0.05) during the active phase (Figure S2b). Crucially, the hallmark signatures of sleep deprivation identified in our study—specifically the robust elevation of S100A3 and the profound depletion of resting NK cells—remained entirely stable and showed no significant differences across the 24‐h cycle.

## 4 Discussion

### 4.1 Research Findings and Implications

This study established an integrative framework that combines transcriptomics with interpretable machine learning to systematically identify and validate biomarkers associated with systemic physiological stress. Unlike conventional approaches that rely solely on differential expression analysis, our multimodel strategy, enhanced by SHAP analysis, ensures both the robustness and interpretability of feature selection. Subsequent validation revealed that S100A3 consistently exhibited diagnostic utility across both acute sleep deprivation and chronic insomnia, whereas VEGFB demonstrated discriminative performance specifically within the chronic context. These findings suggest that S100A3 and VEGFB are not merely sleep‐related markers but pivotal indicators of systemic stress pathophysiology. Consequently, this research offers novel insights into the molecular mechanisms of stress‐induced homeostasis disruption and presents promising candidates for clinical biomarkers, particularly for monitoring systemic inflammatory and metabolic risks associated with sleep disorders.

### 4.2 Biological Function and Toxicological Relevance

#### 4.2.1 Functional Analysis of Characterised Genes

The biomarkers identified in this study, S100A3 and VEGFB, exhibited biological functions congruent with mechanisms frequently implicated in driving systemic physiological stress, thereby establishing a coherent link between sleep deprivation and downstream pathological consequences.

The identification of S100A3 is of particular toxicological importance. As a member of the S100 protein family, S100A3 is associated with damage‐associated molecular patterns (DMAPs), which are released extracellularly in response to cellular stress and tissue injury [31]. By binding to receptors such as RAGE and TLR4, these molecules activate downstream inflammatory pathways, including the NF‐κB pathway [32]. In the context of clinical pathology, DAMPs are well‐established sentinels of stress‐induced tissue injury and sterile inflammation. Although the precise function of S100A3 in sleep remains to be fully elucidated, its marked upregulation under the physiological stress of sleep deprivation suggests it may function similarly to other stress‐responsive S100 proteins, serving as a sensitive circulating indicator of systemic inflammatory stress before overt organ damage occurs.

Furthermore, the involvement of VEGFB aligns with the significant enrichment of the oxidative phosphorylation pathway identified by GSEA. Previous research has demonstrated that VEGFB expression is synergistically regulated by PGC‐1 and ERR‐α, coordinating fatty acid uptake with mitochondrial fatty acid oxidation [33]. Mitochondrial dysfunction is a hallmark mechanism underlying the metabolic dysregulation induced by severe physiological stress. The link between VEGFB and mitochondrial metabolism provides novel insight into how systemic stress may impair energy homeostasis. Additionally, VEGFB has been implicated in neurodegenerative processes, where its elevated expression is associated with neuropathology [34], suggesting that its monitoring could be relevant for assessing neurological risks associated with chronic sleep disruption.

#### 4.2.2 Alterations in the Immune Microenvironment

Our immune infiltration analysis revealed a significant decrease in the proportions of resting NK cells and activated myeloid dendritic cells following systemic stress. Both NK cells and DCs are indispensable for host defence against pathogen invasion and tumorigenesis [35, 36]. The concurrent reduction in these two cell types highlights a multidimensional impairment of the innate immune system [37]. Concurrently, we observed a significant increase in the proportion of immunosuppressive Tregs, alongside an accumulation of naive B cells.

From a clinical immunology perspective, this specific pattern—suppressed innate immunity combined with upregulated regulatory T cells—closely mimics the immunosuppressive profile observed in chronic systemic inflammatory conditions [38, 39]. This state of immune paralysis renders the host susceptible to infections, a common adverse drug reaction. The consistent correlation between S100A3 expression and immune cell shifts reinforces its potential as a core indicator of stress‐induced immunomodulation. Thus, the AI framework presented here captures an immune signature that could be utilised to screen for immune vulnerability and assess the risk of stress‐induced immune dysfunction in patients with sleep disorders.

#### 4.2.3 Differential Expression Threshold Selection

The threshold for differential gene expression in this study was set at |log_2_FC| ≥ 0.3. This threshold was deliberately selected on the basis of the established pathophysiology of sleep deprivation, which, as a perturbation of physiological homeostasis, induces broad yet subtle transcriptomic responses rather than dramatic alterations in gene expression [40]. Research has shown that even short‐term sleep deprivation impacts hundreds of genes, with most changes being modest in magnitude [41]. Consequently, the application of overly stringent thresholds risks excluding key regulatory genes with subtle but biologically meaningful alterations, thereby increasing the rate of false negatives [42]. The adoption of a more sensitive cutoff was therefore a strategic decision aimed at capturing the nuanced and consistent transcriptional changes essential for elucidating the complex molecular networks that govern sleep homeostasis.

#### 4.2.4 Implications for Chronic Disease Monitoring

A central aspect of this study was the validation of biomarkers identified from an acute stress model within an independent chronic stress cohort. Although clinically distinct, acute sleep deprivation and insomnia converge on fundamental molecular pathways, including neuroinflammation [43] and the dysregulation of inflammatory signalling [44]. The successful validation of S100A3 and VEGFB in chronic insomnia substantiates the hypothesis that these markers are robust against the duration of stress exposure. In the context of sleep disorders, physiological stress can be either acute or chronic. The ability of S100A3 to bridge acute and chronic stress models—supported by shared circadian and inflammatory mechanisms [45]—suggests it holds promise as a versatile biomarker for longitudinal clinical monitoring, capable of tracking the progression from acute physiological perturbation to chronic pathological adaptation. Furthermore, a potential confounding factor in our validation strategy is age. The chronic insomnia cohort specifically included elderly patients, whereas the acute sleep deprivation cohorts primarily consisted of younger individuals. It is well‐documented that baseline immune profiles and gene expression naturally shift with age, a phenomenon known as immunosenescence [46]. However, the consistent discriminative power of S100A3 across both the young/acute cohort and the elderly/chronic cohort highlights its remarkable robustness. This cross‐age consistency suggests that S100A3 expression is strongly driven by the overarching physiological stress of sleep deprivation, superseding age‐related baseline variations, thereby reinforcing its utility as a reliable biomarker.

### 4.3 Limitations and Future Prospects

Despite its promising findings, this study has limitations. First and foremost, our study utilised sleep deprivation as a proxy model for systemic stress. While this reveals pathways underlying systemic stress, we did not validate these biomarkers in broader clinical cohorts with overt metabolic or inflammatory diseases. Therefore, it is imperative to validate the identified genes in larger, diverse clinical populations to establish their specificity as stress biomarkers. Secondly, it is important to acknowledge that performing differential gene expression analysis on the entire training cohort prior to cross‐validation may introduce a risk of data leakage. This likely contributed to the perfect classification metrics observed in the Elastic Net and MLP models during the training phase. Future studies should employ nested cross‐validation to rigorously isolate feature selection within the training loops. However, we emphasise that the core validity of the identified biomarkers in this study is strongly supported by their robust diagnostic performance in completely independent external validation cohorts, where such selection bias is structurally precluded. Furthermore, the present analysis relies on bioinformatics predictions without accompanying functional validation. Future work should incorporate in vitro or in vivo stress models to verify the biological functions of the candidate genes and associated pathways. This study was also confined to the transcriptomic level; integrating multiomics data would provide a more comprehensive landscape of the systemic stress response. Collectively, this work establishes a computational foundation for discovering stress‐responsive biomarkers from physiological data.

## 5 Conclusion

In this study, we established and validated a novel analytical framework that integrates multialgorithm machine learning with interpretable AI, enhancing the discovery of robust biomarkers from high‐dimensional physiological stress data. The application of this framework yielded two primary translational insights relevant to clinical monitoring. First, we identified S100A3 as a promising candidate biomarker of systemic physiological stress across diverse contexts. In parallel, VEGFB emerged as a potential indicator of chronic metabolic adaptation, showing significant predictive power in insomnia but not in all acute settings—specifically reflecting sterile inflammation and metabolic adaptation, which are core mechanisms in stress‐induced physiological impairment. Second, we revealed that systemic stress reshapes the immune microenvironment into an immunosuppressive phenotype, a pattern that parallels pathological immune suppression. In conclusion, while based on a physiological model, the computational strategy presented here offers a powerful, non‐invasive approach for dissecting molecular networks of systemic stress. These findings provide a theoretical proof‐of‐concept for utilising interpretable AI to identify potential stress biomarkers capable of monitoring subclinical physiological disruptions and guiding clinical risk stratification in sleep medicine.

## Author Contributions

**Yuming Peng:** project administration. **Junying Zhou:** project administration. **Qiang Zong:** conceptualization, methodology, formal analysis, writing – review and editing. **Chengcheng Wang:** software, visualization, data curation. **Aimin Wu:** funding acquisition, resources. **Peng Wang:** investigation, formal analysis. **Kun Wang:** conceptualization, methodology, software, data curation, validation, writing – original draft, visualization. **Min Wu:** project administration. **Zhenhao Shuai:** supervision, project administration. **Fangfu Ye:** supervision. **Jianwei Shuai:** conceptualization, writing – review and editing, funding acquisition, resources, supervision, formal analysis. **Yanyan Zheng:** funding acquisition, resources.

## Funding

This work is supported by the Ministry of Science and Technology of the People's Republic of China (STI2030‐Major Projects2021ZD0201900), the National Natural Science Foundation of China under Grant U24A2014, the Zhejiang Provincial Natural Science Foundation (LTGY23H090014), the National Key Research and Development Program of China [2020YFC2005605], the Xinjiang Tianshan Talents Project [TSYC202301A076], and the Xinjiang Regional Collaborative Innovation Project [2021E02080]. General Project of Zhejiang Provincial Health Science and Technology Plan (Wenzhou) (2025HY1108) and Major Science and Technology Project of Wenzhou Municipal Science and Technology Bureau (ZY2022024).

## Conflicts of Interest

The authors declare no conflicts of interest.

## Supporting information


**Table S1:** Baseline characteristics of training and validation cohorts. Abbr: SD, sleep deprivation; Exp, experimental group; Con, control group.
**Table S2:** Detailed information on the 25 differentially expressed genes (DEGs). DEGs were identified in the training cohort by comparing sleep deprivation samples to control samples. The screening criteria were an adjusted *p*‐value < 0.05 and |log2FC| ≥ 0.3.
**Table S3:** Optimal parameters for machine learning models.
**Table S4:** Performance evaluation of machine learning models.
**Table S5:** SHAP values of genes in different machine learning models.
**Figure S1:** ROC curves evaluating the diagnostic performance of the remaining six candidate genes. (a) Training cohort, (b) sleep deprivation validation cohort, and (c) insomnia cohort. These genes (RGS18, CLDN7, HPS5, GZMB, ZNF594, COX7B) showed less consistent diagnostic performance across the validation cohorts compared to S100A3 and VEGFB, as shown in the main text.
**Figure S2:** Specificity validation in a normal circadian rhythm cohort. (a) Boxplot showing the stable expression of S100A3 across different physiological states (wakefulness and sleep) over a 28‐h normal circadian cycle. The global ANOVA test indicates no significant time‐of‐day fluctuations. (b) CIBERSORT immune cell fraction analysis comparing the daytime (Blue) and nighttime (Red) phases.

## Data Availability

The data that support the findings of this study are available in GEO at https://www.ncbi.nlm.nih.gov/gds?term, reference number GSE. These data were derived from the following resources available in the public domain:—GSE98582, https://www.ncbi.nlm.nih.gov/geo/query/acc.cgi?acc=GSE98582—GSE37667, https://www.ncbi.nlm.nih.gov/geo/query/acc.cgi?acc=GSE37667—GSE56931, https://www.ncbi.nlm.nih.gov/geo/query/acc.cgi?acc=GSE56931—GSE208668, https://www.ncbi.nlm.nih.gov/geo/query/acc.cgi?acc=GSE208668—GSE48113, https://www.ncbi.nlm.nih.gov/geo/query/acc.cgi?acc=GSE48113. All the datasets were obtained from the GEO (http://www.ncbi.nlm.nih.gov/geo) database. Code is available on https://github.com/wangk‐oj/BioSleep‐AI.
