# Supplementary material for: Integration of Transcriptomics With Interpretable Artificial Intelligence for Identifying Molecular Signatures of Physiological Stress in Sleep Deprivation
Source: J Cell Mol Med. 2026 May 29;30(11):e71211. doi: 10.1111/jcmm.71211 (PMC13240488; doi:10.1111/jcmm.71211)

**Supplementary Materials**

**Table S1** Baseline characteristics of training and validation cohorts. Abbr: SD, sleep deprivation; Exp, experimental group; Con, control group.

| Cohort type | Dataset | Platform | Type | Samples | | Sample  source | Study design |
| --- | --- | --- | --- | --- | --- | --- | --- |
|  |  |  |  | **Exp** | **Con** |  |  |
| Training | GSE98582 | GPL6244 | SD | 27 | 27 | Blood | Con: day 2, 8pm; SD: day 4, 8pm |
|  | GSE37667 | GPL570 | SD | 9 | 9 | Blood | Con: day1, 8am; SD: day3, 8am |
| Validation | GSE56931 | GPL10379 | SD | 13 | 14 | Blood | Con: day1, 8pm; SD: day3, 8pm |
|  | GSE208668 | GPL10904 | Insomnia | 17 | 25 | Blood | N/A |

**Table S2.** Detailed information of the 25 differentially expressed genes (DEGs). DEGs were identified in the training cohort by comparing sleep deprivation samples to control samples. The screening criteria were an adjusted p-value < 0.05 and |log₂FC| ≥ 0.3.

| Gene | logFC | P-Value | change |
| --- | --- | --- | --- |
| COX7B | 0.536886 | 0.000316 | up |
| ZNF594 | 0.421291 | 0.000505 | up |
| EVI2A | 0.374484 | 0.000288 | up |
| ZNF267 | 0.36435 | 0.001052 | up |
| RGS18 | 0.359546 | 8.83E-08 | up |
| RSL24D1 | 0.358811 | 0.003426 | up |
| FMN1 | 0.341111 | 0.021563 | up |
| CAPZA2 | 0.336449 | 0.000255 | up |
| CLDN7 | 0.325533 | 3.75E-06 | up |
| LSM3 | 0.321804 | 0.013315 | up |
| S100A3 | 0.316471 | 2.15E-07 | up |
| TTC32 | 0.313388 | 0.000195 | up |
| SNRPG | 0.307258 | 0.018023 | up |
| RPL34 | 0.305014 | 0.001937 | up |
| GZMK | 0.304751 | 0.024248 | up |
| BCL2A1 | 0.302442 | 0.004685 | up |
| KLRC3 | -0.38123 | 0.017223 | down |
| VEGFB | -0.33751 | 3.05E-06 | down |
| GZMH | -0.33446 | 0.015365 | down |
| PRF1 | -0.33317 | 0.000548 | down |
| GZMB | -0.31907 | 0.00231 | down |
| NCAM1 | -0.31089 | 0.000173 | down |
| HPS5 | -0.3072 | 8.85E-05 | down |
| BIVM | -0.30595 | 0.000982 | down |
| FGFBP2 | -0.30213 | 0.036618 | down |

**Table S3** Optimal parameters for machine learning models.

| Model | Parameters |
| --- | --- |
| SVM | sigma=0.01, C=10 |
| XGBoost | nrounds=200, max_depth=9, eta=0.3, gamma=1, colsample_bytree=0.8, min_child_weight=1, subsample=0.5 |
| RF | mtry=2 |
| ElasticNet | alpha=0.1, lambda=0.01 |
| NB | fL=0, usekernel=TRUE, adjust=0.5 |
| MLP | size=10, decay=0.1 |

**Table S4** Performance evaluation of machine learning models.

| Model | Accuracy | AUROC | Specificity | Precision | Recall | F1-score |
| --- | --- | --- | --- | --- | --- | --- |
| SVM | 0.972 | 1 | 0.971 | 0.975 | 0.975 | 0.973 |
| XGBoost | 0.971 | 0.988 | 0.943 | 0.956 | 1 | 0.975 |
| RF | 0.945 | 0.996 | 0.943 | 0.950 | 0.946 | 0.945 |
| ElasticNet | 1 | 1 | 1 | 1 | 1 | 1 |
| NB | 0.943 | 0.992 | 0.886 | 0.906 | 1 | 0.947 |
| MLP | 1 | 1 | 1 | 1 | 1 | 1 |

**Table S5** SHAP values of genes in different machine learning models.

| Gene | SVM | XGBoost | RF | ElasticNet | NB | MLP | Total |
| --- | --- | --- | --- | --- | --- | --- | --- |
| VEGFB | 0.725931 | 1 | 1 | 0.621447 | 0.916591 | 0.102893 | 4.366861 |
| CLDN7 | 0.917116 | 0.557534 | 0.542012 | 1 | 0.764662 | 0.373695 | 4.155019 |
| S100A3 | 0.826121 | 0.612283 | 0.710413 | 0.473915 | 1 | 0.166517 | 3.78925 |
| ZNF594 | 0.421641 | 0.540968 | 0.55536 | 0.1504 | 0.509175 | 0.482974 | 2.660518 |
| GZMB | 1 | 0.593928 | 0.570824 | 0.066294 | 0.331441 | 0.058723 | 2.621211 |
| RGS18 | 0.308917 | 0.273044 | 0.567531 | 0.198614 | 0.888733 | 0.079704 | 2.316542 |
| HPS5 | 0.024878 | 0.54402 | 0.751161 | 0.077915 | 0.749742 | 0.048941 | 2.196658 |
| COX7B | 0.432247 | 0 | 0.172223 | 0.241687 | 0.247315 | 1 | 2.093472 |
| NCAM1 | 0.661855 | 0.161843 | 0.198683 | 0.236164 | 0.282454 | 0.219813 | 1.760812 |
| TTC32 | 0.249136 | 0.234851 | 0.348621 | 0.078983 | 0.653305 | 0.078424 | 1.64332 |
| BIVM | 0.249051 | 0.224163 | 0.338508 | 0.168208 | 0.283595 | 0.143062 | 1.406588 |
| FMN1 | 0.510729 | 0 | 0.113189 | 0.366934 | 0.10658 | 0.240732 | 1.338164 |
| RPL34 | 0.338464 | 0.3166 | 0.232551 | 0.128217 | 0.066846 | 0.0377 | 1.120377 |
| LSM3 | 0.293281 | 0.176522 | 0.162757 | 0.027693 | 0.268623 | 0.094287 | 1.023162 |
| CAPZA2 | 0.289302 | 0 | 0.096842 | 0.180703 | 0.289831 | 0.049701 | 0.906378 |
| PRF1 | 0.090706 | 0 | 0.261598 | 0.02405 | 0.323868 | 0.126565 | 0.826788 |
| GZMK | 0.371321 | 0 | 0 | 0.14741 | 0.101972 | 0.167709 | 0.788413 |
| BCL2A1 | 0.063 | 0 | 0.137767 | 0.05585 | 0.457232 | 0.046546 | 0.760395 |
| SNRPG | 0.13302 | 0 | 0.038984 | 0.313921 | 0.082123 | 0 | 0.568047 |
| EVI2A | 0 | 0 | 0.12389 | 0 | 0.395599 | 0.044877 | 0.564366 |
| ZNF267 | 0.025399 | 0 | 0.10137 | 0.000213 | 0.361007 | 0.010458 | 0.498446 |
| FGFBP2 | 0.033213 | 0 | 0.173046 | 0.004027 | 0.199546 | 0.077566 | 0.487397 |
| RSL24D1 | 0.012867 | 0.16181 | 0.038631 | 0.006254 | 0.119568 | 0.033814 | 0.372943 |
| KLRC3 | 0.064504 | 0 | 0.101076 | 0 | 0.000084 | 0.057519 | 0.223183 |
| GZMH | 0.00137 | 0 | 0.017699 | 0.017879 | 0.114458 | 0.005099 | 0.156505 |

**Figure S1** ROC curves evaluating the diagnostic performance of the remaining six candidate genes. (a) Training cohort, (b) sleep deprivation validation cohort, and (c) insomnia cohort. These genes (RGS18, CLDN7, HPS5, GZMB, ZNF594, COX7B) showed less consistent diagnostic performance across the validation cohorts compared to S100A3 and VEGFB shown in the main text.


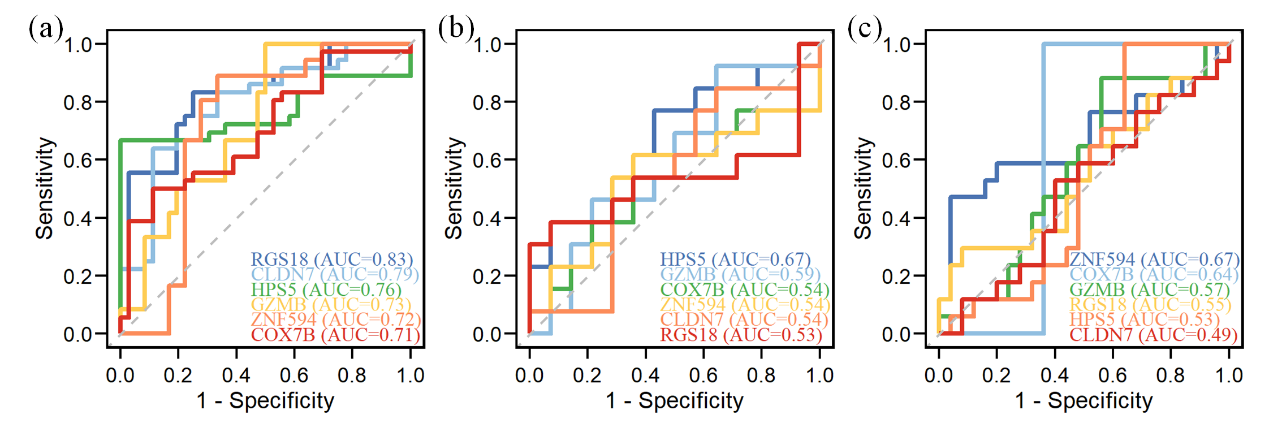


**Figure S2. Specificity validation in a normal circadian rhythm cohort.** **(a)** Boxplot showing the stable expression of *S100A3* across different physiological states (wakefulness and sleep) over a 28-hour normal circadian cycle. The global ANOVA test indicates no significant time-of-day fluctuations. **(b)** CIBERSORT immune cell fraction analysis comparing the daytime (Blue) and nighttime (Red) phases.


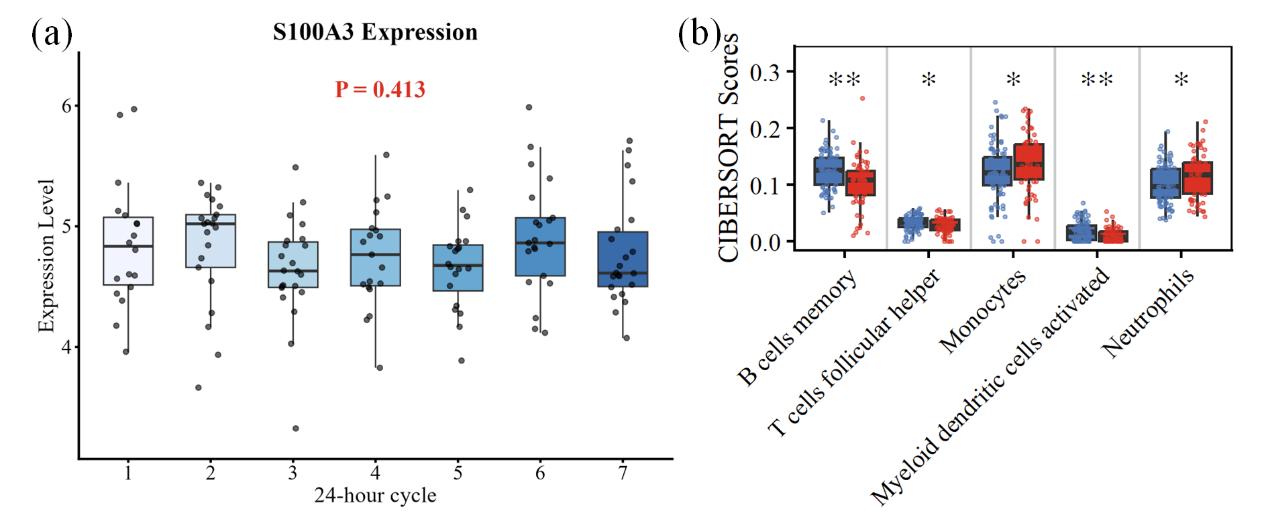

Supplement: Supplementary file 1 — Table S1: Baseline characteristics of training and validation cohorts. Abbr: SD, sleep deprivation; Exp, experimental group; Con, control group. Table S2: Detailed information on the 25 differentially expressed genes (DEGs). DEGs were identified in the training cohort by comparing sleep deprivation samples to control samples. The screening criteria were an adjusted p‐value < 0.05 and |log2FC| ≥ 0.3. Table S3: Optimal parameters for machine learning models. Table S4: Performance evaluation of machine learning models. Table S5: SHAP values of genes in different machine learning models. Figure S1: ROC curves evaluating the diagnostic performance of the remaining six candidate genes. (a) Training cohort, (b) sleep deprivation validation cohort, and (c) insomnia cohort. These genes (RGS18, CLDN7, HPS5, GZMB, ZNF594, COX7B) showed less consistent diagnostic performance across the validation cohorts compared to S100A3 and VEGFB, as shown in the main text. Figure S2: Specificity validation in a normal circadian rhythm cohort. (a) Boxplot showing the stable expression of S100A3 across different physiological states (wakefulness and sleep) over a 28‐h normal circadian cycle. The global ANOVA test indicates no significant time‐of‐day fluctuations. (b) CIBERSORT immune cell fraction analysis comparing the daytime (Blue) and nighttime (Red) phases. [file JCMM-30-e71211-s001.docx]
